# Hyporesponsiveness to the anti-inflammatory action of interleukin-10 in type 2 diabetes

**DOI:** 10.1038/srep21244

**Published:** 2016-02-17

**Authors:** Julianne C. Barry, Soroush Shakibakho, Cody Durrer, Svetlana Simtchouk, Kamaldeep K. Jawanda, Sylvia T. Cheung, Alice L. Mui, Jonathan P. Little

**Affiliations:** 1 School of Health and Exercise Sciences, University of British Columbia Okanagan, Kelowna, BC, Canada; 2 Department of Surgery, University of British Columbia, Vancouver, BC, Canada; 3 Department of Biochemistry and Molecular Biology, University of British Columbia, Vancouver, BC, Canada

## Abstract

Chronic low-grade inflammation contributes to the pathology and complications of type 2 diabetes (T2D). Interleukin-10 (IL10), an anti-inflammatory cytokine, is suggested to play a protective role in T2D. However, the impact of T2D on IL10 function has not been previously assessed. We examined the ability of IL10 to inhibit inflammation in human T2D immune cells and explored underlying mechanisms using macrophage models. IL10 was less effective at inhibiting tumour necrosis factor (TNF)-α secretion in T2D whole blood cultures, which was not explained by altered IL10 receptor surface expression. These findings were observed in macrophages exposed to high glucose, which demonstrated similar IL10 resistance or hyporesponsiveness. These findings were also not explained by changes in IL10 receptor protein or other downstream signaling proteins. High glucose was also shown to impair the ability of IL10 to activate STAT3, a downstream signaling protein of IL10. Treatment with the SHIP1 agonist, AQX-MN100, reversed IL10 hyporesponsiveness in macrophages cultured in high glucose and showed equal effectiveness at different glucose conditions. This data supports the idea that IL10 hyporesponsiveness may contribute to chronic inflammation in T2D. These novel findings suggest that strategies aimed to overcome IL10 hyporesponsiveness may hold therapeutic potential for reducing inflammation in T2D.

Chronic low-grade inflammation plays a critical role in the development and progression of insulin resistance and type 2 diabetes (T2D). The pro-inflammatory environment in T2D is characterized by elevated circulating pro-inflammatory cytokines and acute phase reactants, increased markers of leukocyte activation, and increased macrophage infiltration into adipose and other tissues^1^,^2^. Elevated glucose is the direct metabolic consequence of insulin resistance, and hyperglycemia has been shown to promote cellular inflammation in circulating monocytes^3^,^4^, macrophages^5^, and adipocytes and to contribute to the pathogenesis of T2D and its complications^1^,^2^. Thus, a vicious cycle is created whereby inflammation induces insulin resistance, leading to elevated glucose that can propagate further inflammation. Understanding the mechanisms underlying the pro-inflammatory state in T2D, and how to mitigate it, is therefore of great therapeutic value for the prevention and treatment of T2D and its costly complications.

Most previous research on inflammation in T2D has focused on inhibiting pro-inflammatory signalling and/or neutralizing pro-inflammatory cytokines as therapeutic targets^6^,^7^,^8^. These efforts have revealed mixed results with some studies showing benefits on T2D-related metabolic outcomes^6^,^9^,^10^,^11^, whereas others did not^12^. The role of anti-inflammatory cytokines and signalling in T2D has received much less attention.

Interleukin-10 (IL10) is an anti-inflammatory cytokine normally released locally from immune cells to help resolve inflammation, and is best characterized for its ability to inhibit macrophage activation^13^. Deficiencies in IL10 expression, or IL10 receptor (IL10R) signalling, in mice and humans results in inflammatory diseases such as colitis^14^,^15^,^16^,^17^. There is some evidence suggesting that IL10 may be involved in T2D-related inflammation as mice engineered to ectopically express IL10 through either gene transfer^18^ or through a muscle cell-specific transgene^19^ are partially protected from high fat diet (HFD)-induced obesity and glucose intolerance. However, the impact of T2D on IL10 anti-inflammatory function has not been previously assessed.

IL10 signaling consists of a ligand-specific IL10R1 and a second subunit IL10R2, which is also found in other cytokine receptors^13^. Binding of IL10 to the IL10R activates the STAT3 transcription factor. Our previous research has also shown that activation of the SHIP1 inositol phosphatase is required for IL10 action^20^,^21^. SHIP1 is expressed predominantly in hemopoietic cells and inhibits pro-inflammatory signalling (induced by agents such as bacterial lipopolysaccharide [LPS]) by degrading PIP_3_^22^,^23^. We have also shown that SHIP1-mediated IL10 signaling functions to inhibit translation of TNF-α mRNA, contributing to its anti-inflammatory action^24^. Recent research has also shown that IL10 activates the cellular energy gauge 5′AMP-activated protein kinase (AMPK)^25^,^26^ and that AMPKα1 is required for the anti-inflammatory effects of IL10^26^. Given the emerging role for AMPK as a key integrator of cellular metabolism and inflammation^27^ and studies indicating that high glucose reduces AMPK activation (reviewed in:^28^,^29^), we hypothesized that T2D may impact inflammation by interfering with IL10 signaling. The purpose of this study was to determine how T2D and hyperglycemia influenced the anti-inflammatory abilities of IL10 in immune cells. We examined the ability of IL10 to inhibit inflammatory activation of immune cells in humans with T2D and explored the underlying mechanisms using macrophage cell culture models.

## Results

### IL10 is less effective at inhibiting inflammation in humans with T2D

To determine how T2D *in vivo* impacted IL10 function, we first examined the ability of IL10 to inhibit pro-inflammatory cytokine secretion from whole blood cultures prepared from patients diagnosed with T2D (n = 24, fasting glucose >7.0 mmol/L and/or haemoglobin A1C >6.4%) compared to non-T2D (n = 22) age and body mass index (BMI)-matched controls. Characteristics of the patients, including age, BMI, waist circumference (WC), fasting glucose, and plasma cytokines are presented in Table 1. Patients with T2D had significantly higher fasting plasma glucose (P < 0.001) but were similar age, BMI and WC (all P > 0.05). Continuous glucose monitoring (CGM) was also obtained on a subset of patients with (n = 15) and without (n = 11) T2D. Patients with T2D had a significantly higher 24-hour CGM average blood glucose (7.7 ± 1.8 mmol/L) compared with non-T2D controls (5.8 ± 0.8 mmol/L), confirming that immune cells were exposed to chronically elevated glucose levels (P < 0.05). As expected, the basal pro-inflammatory cytokines TNF-α and IL6 were elevated in patients with T2D (both P < 0.05). Fasting IL10 concentrations were not significantly different (P > 0.05).

**Figure 1:**
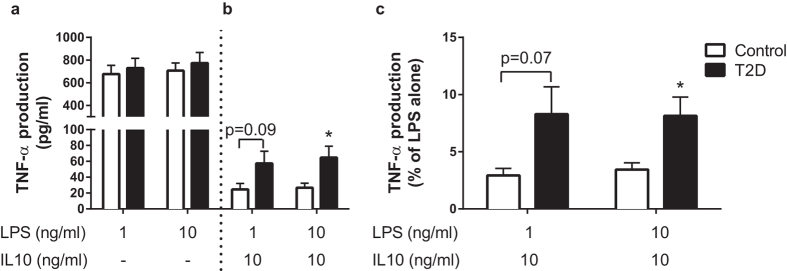
Humans with T2D display hyporesponsiveness to IL10. Whole blood cultures were stimulated with LPS only (1 and 10 ng/ml) or LPS + IL10 (10 ng/ml) and pro-inflammatory activation was assessed by TNF-α secretion. A two-way ANOVA revealed no significant differences in (**a**) LPS-induced TNF-α secretion between T2D patients (n = 24) and age and BMI-matched non-T2D controls (n = 22) at both 1 ng/ml and 10 ng/ml LPS. However, the ability of IL10 to suppress TNF-α secretion was less effective in T2D (main effect of group, P < 0.05) for both absolute (**b**) and % maximal (**c**) TNF-α secretion with post-hoc tests revealing a significant difference at 10 ng/ml LPS combined with 10 ng/ml IL10 (P < 0.05 for both). Biological replicates were cultured and TNF-α secretion measured by standard ELISA. IL10 inhibition is expressed as % maximal TNF-α secretion within each subject calculated as ([(LPS-induced secretion–LPS + IL10 secretion)/LPS secretion] × 100%). Values are mean ± SEM, *P < 0.05 vs. Control.

Whole blood cultures were used to minimize any impact of sample processing and maintain physiological conditions of plasma constituents and cellular composition^30^. Cultures were stimulated with LPS (1 and 10 ng/ml) +/− IL10 (10 ng/ml). A two-way ANOVA revealed a significant main effect of LPS (P = 0.047, as expected) with no significant difference between T2D and non-T2D (main effect of group, P = 0.623; Fig. 1a). As described previously^20^,^21^,^31^, the ability of IL10 to inhibit LPS-induced TNF-α secretion was used as an index of the anti-inflammatory ability of IL10. IL10 inhibited LPS-induced TNF-α secretion with results of the two-way ANOVA indicating that the anti-inflammatory action of IL10 was lower in T2D vs. non-T2D control participants (main effect of group, P ≤ 0.05, Fig. 1). The findings were similar when TNF-α was expressed as an absolute concentration (P = 0.05; Fig. 1b) or as a percentage of maximal LPS-induced secretion (P = 0.032; Fig. 1c), with post-hoc testing revealing a significant difference (P < 0.05) between T2D and non-T2D in the 10 ng/ml LPS + 10 ng/ml IL10 condition. Secretion of IL10 from whole blood cultures of T2D patients and non-T2D controls at both 1 ng/ml LPS (T2D: 4.5 ± 3.5 ng/ml; Non-T2D: 5.6 ± 4.9 ng/ml) and 10 ng/ml LPS (T2D: 4.7 ± 3.5 ng/ml; Non-T2D: 5.4 ± 3.9 ng/ml) were not significantly different (P > 0.05).

**Figure 2:**
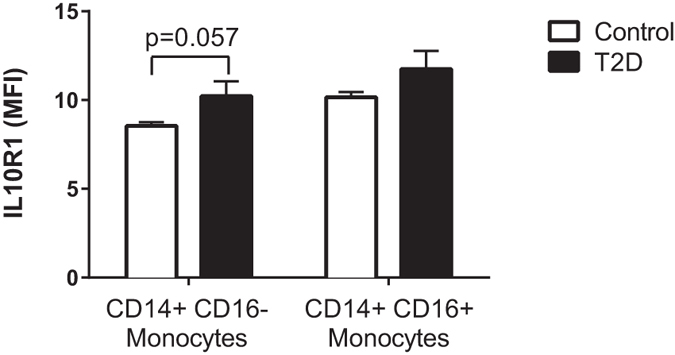
No significant difference in IL10R1 expression on monocytes in T2D. IL10R1 was measured on CD14+/CD16− and CD16+ monocytes by flow cytometry in the T2D and age and BMI-matched non-T2D control participants. Unpaired t-tests indicated that IL10R1 expression was not significantly different on either monocyte subset although IL10R1 tended to be lower on CD14+/CD16− classical monocytes in T2D (P = 0.057). Values are mean ± SEM.

Monocytes, the precursors to tissue macrophages, are the major cell type that produce pro-inflammatory cytokines (including TNF-α), in whole blood cultures^32^,^33^. In order to determine whether reduced expression of the IL10R might explain the lower anti-inflammatory function of IL10 in patients with T2D we performed flow cytometry experiments to analyze the surface protein expression of the IL10R1 on “classic” CD14+/CD16- monocytes and “pro-inflammatory” CD14+/CD16+ monocytes. We analyzed monocyte subsets because evidence indicate that CD16+ monocytes produce the majority of TNF-α in human whole blood^32^ and data suggesting that IL10 expression may be different in monocyte subsets^34^. There were no significant differences in IL10R1 detected (Fig. 2), but there was a trend (P = 0.057) for an increase in median fluorescence intensity of IL10R1 on classical CD14+/CD16− monocytes in T2D.

Thus, immune cells from individuals with T2D appear to be resistant to the anti-inflammatory effects of IL10, which are not explained by reductions in the surface protein expression of IL10R1.

### High glucose promotes IL10 resistance in macrophages

**Figure 3:**
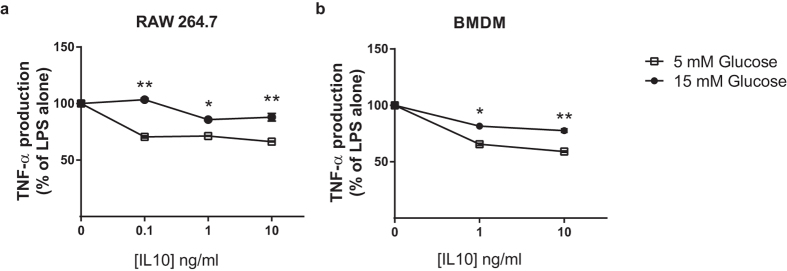
RAW264.7 cells and BMDMs (bone marrow-derived macrophages) grown in 15 mM glucose are hyporesponsive to IL10. RAW264.7 cells (**a**) and BMDMs (**b**) were cultured for six hours in 9% FCS in DMEM containing either 5 mM or 15 mM glucose. Cells were then stimulated for two hours with 1 ng/mL LPS in the presence of 0–10 ng/mL of IL10, and supernatants collected for TNF-α production (expressed as % of that induced by LPS alone) by ELISA. Values are mean ± SEM (N = 3). *p < 0.05 & **p < 0.001 vs. 5 mM glucose condition.

In obesity and T2D, blood monocytes are recruited into tissues (e.g. adipose, liver) where they differentiate into macrophages and propagate a state of chronic low-grade inflammation^1^. In order to determine if hyperglycemia, the primary pathophysiological hallmark of T2D, might be responsible for reducing the anti-inflammatory function of IL10, we cultured RAW264.7 mouse macrophages in normal (5 mM) and high (15 mM) glucose and treated the cells with LPS +/− IL10. Similar to the findings in T2D patients, six hour exposure of macrophages to high glucose induced a state of IL10 resistance; IL10 was less effective at inhibiting LPS-induced TNF-α secretion at 15 mM glucose compared to 5 mM glucose (Fig. 3a) in RAW264.7 mouse macrophages. Findings were confirmed in primary mouse bone-marrow derived macrophages (BMDMs) (Fig. 3b).

### High glucose inhibits IL10-mediated STAT3 activation

**Figure 4:**
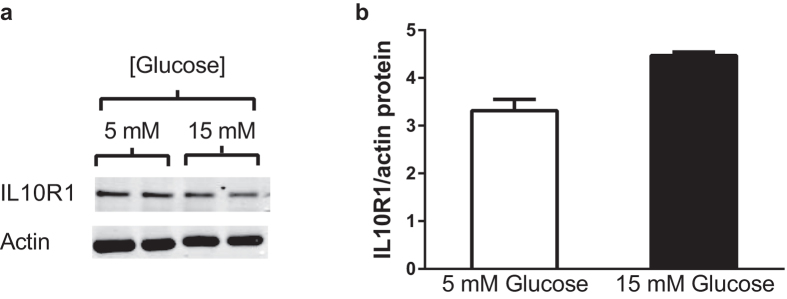
Protein levels of IL10R1 in macrophages are not affected by altered glucose levels. (**a**) Cell lysates from RAW264.7 cells cultured in 5 mM or 15 mM glucose for six hours were immunoblotted with the indicated Abs. Representative immunoblots from duplicates are shown. (**b**) Band intensities were quantified on a Li-Cor Odyssey scanner and normalized to the levels of actin and plotted in the bar graph. Paired t-tests revealed no significant differences between 5 mM and 15 mM glucose. Values are mean ± SEM (N = 3). P > 0.05.

**Figure 5:**
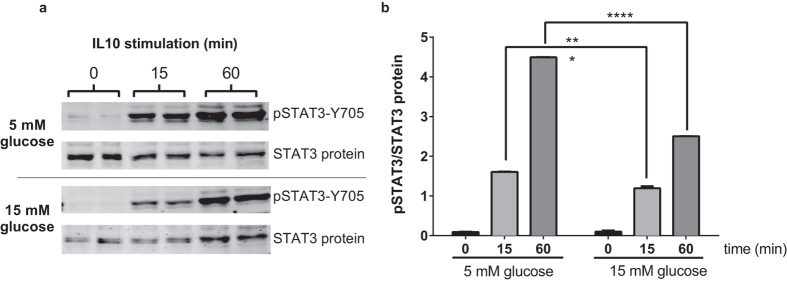
High glucose (15 mM) treatment reduces IL10’s ability induce STAT3 Tyr 705 phosphorylation. RAW264.7 cells were cultured for six hours in either 5 mM or 15 mM glucose. Duplicate sets of cells were then stimulated with 1 ng/mL IL10. (**a**) Cell lysates were separated by SDS-PAGE gel and immunoblotted with antibodies to P-STAT3 Y705 or total STAT3 protein. (**b**) Band intensities were quantified on a Li-Cor Odyssey scanner and P-STAT3/STAT3 protein ratios are shown in the bar graph. Values are mean ± SEM (N = 3). ***P < 0.01, ****P < 0.001 vs. 5 mM glucose condition.

**Figure 6:**
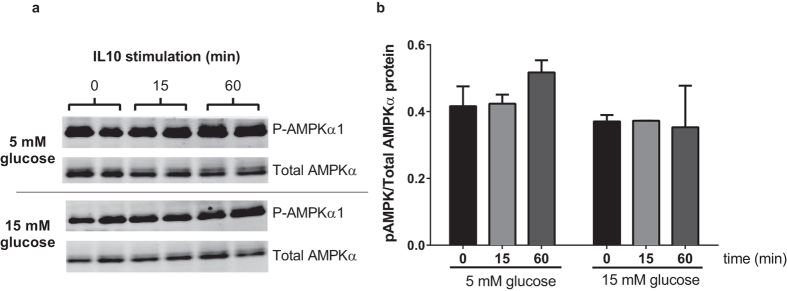
P-AMPK levels are not affected by IL10 treatment in RAW264.7 cells. RAW264.7 cells were cultured for six hours in either 5 mM or 15 mM glucose. Duplicate sets of cells were then stimulated with 1 ng/mL IL10. (**a**) Cell lysates were separated by SDS-PAGE gel and immunoblotted with antibodies to P-AMPKα (Thr-172) and total AMPKα protein. (**b**) Band intensities were quantified on a Li-Cor Odyssey scanner and P-AMPKα/total AMPKα protein ratios shown in the bar graph (no effects of time or condition, all P > 0.05) Values are mean ± SEM (N = 3).

To explore whether high glucose altered IL10 signaling, we first examined the total protein content of the key IL10 signaling component IL10R1 in RAW264.7 macrophages cultured for six hours in 15 mM compared to 5 mM glucose (Fig. 4a). Quantification of immunoblots revealed no significant effects of high glucose on IL10R1, total STAT3, or total AMPKα protein levels (Figs 4b, 5b and 6b). Thus, similar to humans with T2D, hyporesponsiveness to IL10 as a result of hyperglycemia in cultured macrophages did not appear to be explained by reduced protein levels of the IL10 receptor or downstream signalling proteins. We next explored how exposure to high glucose influenced activation of IL10 signaling in macrophages. As expected, treatment of RAW264.7 macrophages treated with IL10 (10 ng/ml) in 5 mM glucose led to a robust activation of STAT3 as assessed by tyrosine-705 phosphorylation (Fig. 5). When RAW264.7 cells were cultured in 15 mM glucose the ability of IL10 to induce STAT3 phosphorylation was significantly reduced (P < 0.001). These findings indicated that high glucose impairs the ability of IL10 to activate intracellular STAT3.

We also examined IL10’s ability to induce phosphorylation of AMPKα at threonine-172 as a marker of activation. Figure 6 shows that phospho-AMPKα levels appear constitutively higher in cells grown in 5 mM than 15 mM glucose, although this did not reach statistical significance. There appeared to be a marginal increase in threonine-172 phosphorylation of AMPKα in response to IL10 treatment in 5 mM glucose conditions, but this was not statically significant. We were not able to detect an increase phosphorylation of AMPKα1 in response to IL10 treatment in cells cultured in 15 mM glucose.

### The SHIP1 agonist AQX-MN100 can overcome high glucose-induced IL10 resistance

**Figure 7:**
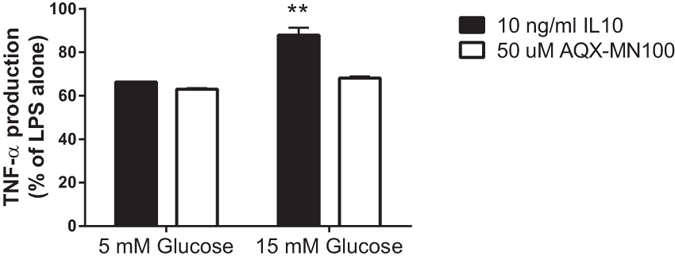
AQX-MN100 can bypass IL10 hyporesponsiveness to inhibit inflammation in cells grown in high glucose. Cells were cultured for six hours in 9% FCS in DMEM containing either 5 mM (Low) or 15 mM (High) glucose. Cells were then stimulated for two hours with 1 ng/mL LPS in the presence of 10 ng/mL IL10 or 50 uM AQX-MN100. TNF-α production was assessed by ELISA. IL10 was less effective in high glucose whereas the anti-inflammatory actions of AQX-MN100 were not influenced. Values are mean ± SEM (N = 3). **p < 0.001 as compared to low glucose.

Given the importance of SHIP-1 in mediating the anti-inflammatory actions of IL10^20^, we next determined whether the small molecule SHIP1 agonist AQX-MN100^35^ was able to overcome IL10 hyporesponsiveness in macrophages cultured under high glucose conditions. Despite the cells being resistant to the anti-inflammatory effects of IL10 in 15 mM glucose, AQX-MN100 was equally effective at inhibiting LPS-induced TNF-α secretion in 15 mM and 5 mM glucose conditions (Fig. 7) demonstrating the ability of a SHIP1 agonist to act as an anti-inflammatory agent under hyperglycemic conditions.

## Discussion

In this study we provide evidence from both human and cell culture studies that T2D *in vivo* and hyperglycemia *in vitro* are related to a reduced anti-inflammatory function of IL10. The hyporesponsiveness to IL10 in the presence of high glucose appears linked to reduced intracellular signal transduction through STAT3, whereas the anti-inflammatory actions of AQX-MN100, a small molecule activator of SHIP1, is not affected by hyperglycemia. Collectively, the results support the novel idea that chronic low-grade inflammation in T2D might be explained, at least in part, by a reduction in the natural anti-inflammatory actions of IL10. Strategies aimed to overcome IL10 resistance, including SHIP1 agonists, might therefore hold therapeutic potential for reducing inflammation associated with metabolic disease.

Previous studies examining a role for IL10 in T2D-related pathology have produced mixed results. Hong *et al.* demonstrated that muscle-specific overexpression of IL10 protected mice from HFD-induced insulin resistance and inflammation^19^. Similarly, Gao *et al.* demonstrated that treatment with IL10 though plasmid injections preserved insulin sensitivity and prevented glucose intolerance in mice fed a HFD^18^. Both of these studies provide evidence that artificially elevating IL10 to supraphysiological levels may have beneficial effects on T2D-related metabolic control, but they were not designed to address the function of IL10 in the context of established T2D. Kowalski *et al.* used bone marrow transfer techniques to create hematopoietic-cell restricted deletion of IL10 in mice and reported no impact of IL10 deletion on HFD-induced inflammation or insulin resistance, arguing against a role for IL10 in T2D-related pathology^36^. However, deletion of IL10 in hematopoietic cells may not be representative of subtle changes in IL10 action that may occur in T2D. We aimed to study how IL10 functioned to inhibit inflammation in immune cells in the context of T2D and hyperglycemia. Our results indicate that the normal anti-inflammatory actions of IL10 are impaired in T2D, effects that appear to be linked to hyperglycemia. These data suggest that the metabolic consequences of T2D may perpetuate a state of chronic low-grade inflammation through a mechanism involving failure of IL10 to adequately resolve innate immune activation.

We are aware of two previous studies linking reduced anti-inflammatory actions of IL10 to chronic inflammation. Avdiushko *et al.* demonstrated that cultured macrophages isolated from mice chronically infected with the LP-BM5 retrovirus had reduced capacity to response to IL10 as assessed by reduced ability of IL10 to inhibit LPS-induced cytokine secretion^37^. This hyporesponsiveness to IL10 upon viral infection was not related to reductions in mRNA expression of the IL10 receptor and the authors were unable to decipher the potential mechanisms responsible but concluded that the chronic inflammatory environment promoted by the LP-BM5 retroviral infection rendered macrophages less responsive to IL10 via a mechanism that was downstream of the IL10 receptor. Yuan *et al.* also found that monocytes isolated from patients with systemic lupus erythematosus (SLE) were less responsive to IL10, assessed by reduced ability of IL10 to suppress human Ig-induced TNF-α and IL6 secretion^38^. The IL10 hyporesponsiveness coincided with reduced IL10-induction of STAT3 phosphorylation in the presence of human Ig, while IL10R and STAT3 expression levels were the same in control and SLE monocytes. Our findings indicate that exposure to high glucose can also reduce the capacity of macrophages to respond to IL10, linking hyperglycemia in T2D to IL10 hyporesponsiveness. Our findings in cultured macrophages show that hyperglycemia impacts IL10 signaling at the level of STAT3, a key node in intracellular IL10 signal transduction^39^. IL10 has been reported to induce phosphorylation of AMPKα1 in primary bone marrow derived macrophages culture^25^,^26^. IL10 was not able to inhibit TNF-α mRNA induction in AMPKα1 knockout macrophages^25^,^26^. Interestingly, AMPKα1 deficiency results in elevated LPS-induced TNF-α levels regardless of the presence of IL10^25^,^26^. This suggests that AMPKα1 might be basally active even in the absence of IL10, and its presence may restrain LPS action. In fact, AMPKα1 activation state can be controlled by many signals including low glucose levels^40^. In our hands we saw no clear effects of IL10 on AMPK using threonine-172 phosphorylation as a marker of AMPKα1 activation. IL10 led to marginal but non-significant AMPK activation in low glucose conditions and no impact in high glucose (Fig. 6b). It is possible that the higher basal levels of phospho-AMPKα1 and elevations in total AMPKα1 protein when cells were cultured in low glucose (Fig. 6a) may have masked any potential enhancing effects of IL10 treatment.

AQX-MN100 is a small molecular activator of SHIP1 that has previously been shown to mimic the anti-inflammatory actions of IL10^21^,^35^. Despite showing impaired ability to respond to IL10 under high glucose conditions, AQX-MN100 was equally effective at inhibiting LPS-induced TNF-α secretion from macrophages grown in high or low glucose concentration. Thus, high glucose does not impair the intrinsic ability of SHIP1 to inhibit inflammation but the ability of IL10 to activate SHIP1 appears to be impaired.

Our results show that IL10 hyporesponsiveness exists in humans with T2D compared to age and BMI-matched non-T2D controls; however, the exact mechanisms contributing to this effect *in vivo* cannot be determined. The parallel cell culture experiments support the notion that hyperglycemia is involved but it is important to acknowledge that differences in medications or other factors between the T2D patients and the non-T2D controls may have impacted the human experiments. Approximately half of the individuals with T2D included in this study (11 out of 24) were taking glucose-lowering medications (either metformin or a combination of metformin with one other drug; see Table 2). Exactly how these diabetes medications interact with hyperglycemia to impact IL10 function is not known. However, we feel that studying individuals on glucose lowering (anti-diabetic) and other medications is more representative of a typical T2D patient. As well, patients did not take their metformin or other drugs on the morning of the fasted blood samples. Thus, the findings of altered IL10 function in individuals with T2D, who displayed evidence of chronic low-grade inflammation based on plasma TNF-α and IL6 levels, suggests that IL10 resistance may have clinical relevance across a diverse group of T2D patients. We were not adequately powered to compare differences between T2D patients on different medications in this study but it may be interesting to explore potential interaction between T2D medications and IL10 function in future research. Another limitation of the current study is that we focused on the impact of elevated glucose for the macrophage culture experiments and results were based on N = 3 independent experiments performed in duplicate. Future studies exploring how other factors that may be elevated in T2D (e.g., lipids, insulin) impact IL10 responsiveness may provide further insight.

## Conclusion

The present findings indicate that IL10 hyporesponsiveness or “IL10 resistance” occurs in immune cells from humans with T2D and in macrophages cultured in physiologically-relevant hyperglycemia. Hyporesponsiveness to IL10 does not appear to be mediated by downregulation of the IL10 receptor but there is impaired downstream IL10 signaling under hyperglycemic conditions that can be overcome with the small molecule SHIP1 agonist AQX-MN100. This is the first study to our knowledge implicating diminished anti-inflammatory IL10 functioning in T2D. A more thorough understanding of potential dysfunction in the IL10 signaling pathway in T2D is required to identify key mechanisms and uncover potential therapeutic options that target relieving IL10 resistance in T2D.

## Methods

### Human participants and experimental methods

#### Participants

Individuals with T2D were recruited via poster advertisement at local medical laboratories, Internet message board advertisements, and word-of-mouth. Age and BMI-matched non-T2D controls were recruited through Internet message boards and word-of-mouth. Sample size was calculated based on IL10-mediated TNF-α inhibition pilot studies conducted in whole blood cultures in our lab that demonstrated a large effect size between T2D and non-T2D controls (~1 SD difference) (n = 7–8 per group). With 80% power and alpha of 0.05, sample size required was estimated to be n = 16 per group (calculated using G*Power Version 3). To preserve power we aimed to recruit 20–25 per group. The study and experimental protocols were approved by the UBC Clinical Research Ethics Board. All procedures were conducted in accordance to approved protocols and all participants provided written informed consent. T2D patients were not on exogenous insulin and any interested participants were excluded if they reported regularly taking anti-inflammatory medications or had a current or recent self-reported infection. Thirteen T2D patients were not taking any anti-diabetic medications and eleven were taking anti-diabetic medications (six metformin, three combination of metformin and sulphonylureas, one combination of metformin and DPP-4 inhibitor, and one combination of metformin and GLP-1 receptor agonist). Ten T2D patients and three non-T2D controls were taking anti-hypertensive medications. Details of medications in the T2D patients are shown in Table 2.

#### Anthropometrics

Body mass, height (Seca 700 Mechanical Colum Scale) and waist circumference (WC; measured at the top of the iliac crest) were measured and BMI was calculated as body mass in kilograms divided by height in meters squared.

#### Blood sampling

Fasting venous blood samples were obtained by venipuncture from an antecubital vein and collected into a sodium heparin and an EDTA tube (BD Vacutainer). Blood collected in the sodium heparin tube was used for whole blood cultures. Plasma was collected by centrifugation of the EDTA tube at 1550 × g for 15 min at 4 °C and stored at −80 °C for further analysis.

Plasma glucose was measured in duplicate using the hexokinase method (G5717-120: Pointe Scientific INC, USA) on a Chemwell 2910 automated analyzer (Awareness Technologies, Palm City, USA). Plasma cytokines (IL10, IL6 and TNF-α) were measured by custom multiplex immunoassay (Human High Sensitivity T Cell multiplex kit: HSTCMAG-28SK, Millipore, Billerica, MA, USA) and read using a MAGPIX™ Bio-Plex® reader (BioRad, Hercules, California, USA) according to the manufacturer’s instructions. Plasma was spun at 1000 × g for 15 min at 4 °C to remove any precipitates and the assay was performed in duplicate. Plasma cytokine concentrations were calculated using a 5-parameter logistic fit on Bio-Plex Manager™ 6.1 Software.

#### Continuous glucose monitoring

Real-time continuous glucose monitoring was measured over 48–72 hours using the Guardian® REAL-Time CGMS and Enlite® sensor (Medtronic MiniMed, Northridge, CA). Sensors were inserted by a trained researcher and participants were instructed on how to obtain finger prick blood samples (OneTouch Ultra Mini) for calibration at least three times per day. Upon device removal, data was downloaded to CareLink Personal Therapy Management Software for Diabetes and 24-hour average glucose was calculated after exporting the data to Microsoft Excel.

#### Human whole blood culture

Whole blood was diluted 1:10 in serum-free RPMI media (Sigma) containing 5 mM glucose with penicillin (50 U/ml) and streptomycin (50 ug/ml). Diluted whole blood (540 ul) was seeded in 24-well plates (Costar). Whole blood cultures were stimulated with lipopolysaccharide (LPS, from *Escherichia coli* 055:B5; L6529, Sigma) at 1 or 10 ng/ml with or without 10 ng/ml IL10. Whole blood cultures were incubated at 37 °C in 5% CO_2_ and supernatants were collected at six hours for analyses of secreted TNF-α on duplicate samples by ELISA (Human TNF-α DuoSet, R&D Systems) according to the manufacturer’s instructions. Absorbance was read at 450 nm on an iMark Microplate Absorbance reader (Biorad, CA, USA) and data were analyzed using Microplate Manager 6.0 (Biorad) to calculate TNF-α concentration using a cubic spline regression model. TNF-α concentration was expressed in pg/ml or as percent of maximal LPS-stimulated secretion.

#### Flow cytometry

IL10 receptor 1 (IL10R1) levels were measured on different monocyte subsets in human whole blood by flow cytometry. 8 ul of FcR Blocking Reagent (Miltenyi, Bergisch Gladbach, Germany) was added to 72 ul of whole blood and allowed to incubate for 10 minutes at 4 °C in the dark. Following this, 2 ul of CD14-Vioblue (Miltenyi, Bergisch Gladbach, Germany), CD16-FITC (Miltenyi, Bergisch Gladbach, Germany), and CD210/IL10R-PE (BioLegend, San Diego, CA) were added and again allowed to incubate for 10 minutes at 4 °C in the dark. After incubation, 1 ml of red blood cell lysis buffer (Miltenyi, Bergisch Gladbach, Germany) was added and the sample was incubated for 15 minutes at room temperature in the dark. In order exclude dead cells from analysis, 2 ul of Propidium Iodide (Miltenyi, Bergisch Gladbach, Germany) was added immediately before analyzing on a MACSQuant Analyzer flow cytometer (Miltenyi, Bergisch Gladbach, Germany). A total of 15000 CD14+ events were collected for analysis in each subject. Events positive for PI were excluded from analysis using a not-gate. The different monocyte subtypes were identified using light scatter characteristics as well as being positive for CD14-Vioblue but negative for CD16-FITC (CD14+/CD16− “classic” monocytes) or positive for both CD14-Vioblue and CD16-FITC (CD16+ “pro-inflammatory” monocytes). Fluorescence minus one (FMO) controls were used to establish positive staining. IL10R1 expression, expressed as median fluorescence intensity, was then analyzed on these monocyte subtype populations.

### Cell culture models and experimental methods

#### Macrophage cell culture

Culture of RAW264.7 cells and derivation of bone marrow derived macrophages were as described^24^,^41^. DMEM of various glucose concentration were generated by mixing appropriate proportions of DMEM high glucose (Hyclone SH30022.01) and DMEM low glucose (Hyclone SH30021.01). Stimulations with LPS and measurement of TNF-α production by ELISA were also as described^24^,^41^.

#### Immunoblotting

Preparation of cell lysates for sodium dodecyl sulphate-gel polyacrylamide gel separation and immunoblot analysis were as described^24^,^41^. Antibodies to pSTAT3-Y705, STAT3 protein, pAMPKα-Thr172, and pAMPKα were from Cell Signalling Technologies (Whitby, Ontario, Canada). Antibodies to murine IL10R1 and SHIP1 (clone P1C1) were from Santa Cruz Biotechnology (Texas, USA).

#### Statistical Analysis

Data were analyzed using SPSS Statistics. Normality of data was assessed using Q-Q plots and the Shapiro-Wilk test and variables that did not meet the assumptions of normality were log transformed prior to statistical analyses. Three outliers were removed from analyses of plasma cytokines based on values that were deemed non-physiological being >10 SD from the mean (one T2D for IL10 and one non-T2D for IL6) and >4 SD from the mean (one non-T2D for IL6). Removal of these outliers from the select plasma cytokine analyses did not change the statistical outcomes but due to their influence on the mean values, were omitted from the analyses and descriptive data. Differences between T2D patients and non-T2D controls were analyzed by independent t-tests. A two-factor (group X concentration) repeated measures ANOVA were used to compare changes in whole blood culture cytokine secretion and for cell culture experiments. Significant main effects or interactions were followed by Fisher LSD post-hoc testing. Statistical significance was set at p ≤ 0.05.

## Additional Information

**How to cite this article**: Barry, J. C. *et al.* Hyporesponsiveness to the anti-inflammatory action of interleukin-10 in type 2 diabetes. *Sci. Rep.* **6**, 21244; doi: 10.1038/srep21244 (2016).

## Figures and Tables

**Table 1:** Characteristics of the T2D patients and non-T2D control participants.

Descriptive Variable	T2D (n = 24)	Non-T2D (n = 22)	p-value
Gender (M/F)	8/16	4/18	
Age (years)	57.8 (10.9)	53.4 (10.7)	0.173
Body mass index (kg/m^2^)	32.1 (6.2)	30.1 (4.8)	0.233
Waist circumference (cm)	109.1 (19.4)	102.3 (13.5)	0.183
Fasting plasma glucose (mmol/L)	7.8 (0.8)*	5.8 (0.8)	**<0.001**
24 Hour CGM average glucose (mmol/L)	7.7 (1.8)*	5.8 (0.8)	**0.003**
Plasma TNF-α (pg/ml)	9.57 (5.98)*	5.75 (2.72)	**0.009**
Plasma IL6 (pg/ml)	3.02 (2.27)*	1.76 (0.94)	**0.049**
Plasma IL10 (pg/ml)	7.07 (5.93)	4.52 (3.99)	0.163

T2D patients (n = 24) exhibit elevated fasting TNF-α (P = 0.009) and IL6 (P = 0.049) but no significant difference in fasting IL10 (P = 0.163) versus non-T2D control participants (n = 22). All values are mean (SD).

**Table 2:** Medication usage in T2D patients.

Medication usage in T2D patients	(n = 24)
Anti-Diabetic (No.)	
Biguanide	6
Biguanide + Sulphonylurea	3
Biguanide + GLP-1R antagonist	1
Biguanide/DPP-4 inhibitor	1
Anti-hypertensive (No.)	
Calcium channel blocker	2
ACE inhibitor	1
Angiotensin II receptor blocker	2
Beta blocker	1
Calcium channel blocker + ACE inhibitor	2
Calcium channel blocker + Angiotensin II receptor blocker	1
Angiotensin II receptor blocker + Beta blocker	1

The two main medication classes used in the T2D patients were anti-diabetic medications (11 out of 24) and anti-hypertensive medications (10 out of 24).
